# Asymptomatic pulmonary penicilliosis with a lung mass in an HIV‐infected patient

**DOI:** 10.1002/jgf2.325

**Published:** 2020-05-13

**Authors:** Masashi Nishikubo, Asako Doi, Hiroshi Takegawa, Daisuke Yamashita, Junichiro Ohira, Hiroaki Nishioka

**Affiliations:** ^1^ Department of General Internal Medicine Kobe City Medical Center General Hospital Kobe Japan; ^2^ Department of Infectious Disease Kobe City Medical Center General Hospital Kobe Japan; ^3^ Department of Laboratory Medicine Kobe City Medical Center General Hospital Kobe Japan; ^4^ Department of Pathology Kobe City Medical Center General Hospital Kobe Japan; ^5^ Department of Neurology Kobe City Medical Center General Hospital Kobe Japan

**Keywords:** asymptomatic respiratory infection, HIV, lung mass, *Penicillium marneffei*

## Abstract

*Penicillium marneffei* (*Talaromyces marneffei*) infection sometimes occurs in HIV‐infected patients in South‐East Asia. However, reports on asymptomatic cases are rare. Herein, we report a case of a 27‐year‐old Burmese HIV‐positive woman with pulmonary penicilliosis. Chest radiography showed a lung mass; however, the patient did not have any respiratory symptoms. Cultures of bronchoalveolar lavage and lung tissue grew *P marneffei*. The patient was diagnosed with penicilliosis and successfully treated with amphotericin B and itraconazole. Our findings suggest that *P marneffei* infection should be considered in the differential diagnosis of a lung mass in HIV‐infected patients, even when asymptomatic for respiratory symptoms.

## Graphical abstract

We report the case of pulmonary penicilliosis, which showed a lung mass on chest radiography but without respiratory symptoms. Reports on asymptomatic cases are rare.

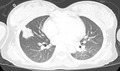

## 1 INTRODUCTION

*Penicillium marneffei* (*Talaromyces marneffei*) infection is an important cause of morbidity and mortality in HIV‐infected patients, with a high prevalence in South‐East Asia.^1^ The respiratory system is commonly infected in penicilliosis. Patients with pulmonary penicilliosis usually have respiratory symptoms such as cough, sputum, dyspnea, or chest pain,^2^ with abnormal chest radiography findings including plural effusion, alveolar consolidation, nodule lesions, miliary lesions, or cavitary lesions.^3^, ^4^ Asymptomatic cases of penicilliosis are rarely reported. Herein, we report on a patient with asymptomatic pulmonary penicilliosis presenting a lung mass.

## 2 CASE REPORT

**Figure 1:**
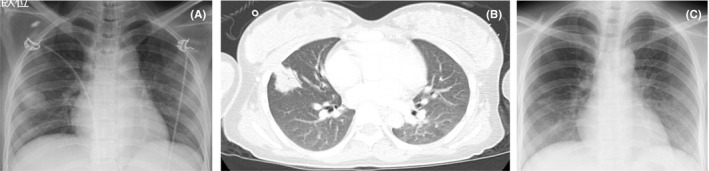
A, Chest radiograph, showing a 2.1 × 2.6 cm^2^ nodule in the right lung (white arrowheads). B, Chest computed tomography, showing a 2.6 × 2.7 cm^2^ irregular nodule with air bronchogram in the right S4 (white arrowheads). C, Chest radiograph, showing the diminution of the lung nodule 30 d after treatment initiation

The patient, a 27‐year‐old Burmese woman, had married a Japanese man and came to Japan one month prior to admission. She was in her 31st gestational week and received antenatal care in Yangon, Myanmar, where no problems were identified. One week prior to admission, she started experiencing a headache without any other symptoms. On the day of admission, her husband observed that she had consciousness disturbance and brought her to our hospital. Physical examination showed that her blood pressure was 103/75 mm Hg, pulse rate 113 beats/min, respiratory rate 18 breaths/min, and body temperature 37.8°C. We assessed her consciousness as E4V1M5 on the Glasgow Coma Scale. Examination of her respiratory, cardiovascular, and gastrointestinal systems, and skin revealed unremarkable findings. Laboratory findings showed a white cell count of 5200/μL (neutrocytes 78%), a hemoglobin level of 11.5 g/dL, a platelet count of 25.8 × 10^4^/μL, a C‐reactive protein level of 0.57 mg/dL, and beta‐d‐glucan under 6.0 pg/mL, and aspergillus galactomannan antigen test was negative. HIV infection was confirmed based on positive serology. Her CD4 T‐cell count was 18/μL, and HIV‐1 RNA load was 1.2 × 10^5^ copies/mL. Chest radiography showed a 2.1 × 2.6 cm^2^ nodule in the right middle lobe of the lung (Figure 1A), and chest computed tomography also revealed a 2.6 × 2.7 cm^2^ irregular nodule with an air bronchogram in the right middle lobe (S4) (Figure 1B). Head computed tomography showed hydrocephalus. We suspected a toxoplasma encephalopathy, which was later confirmed by positive serum anti‐*Toxoplasma gondii* IgG antibody findings, as well as the detection of *Toxoplasma* DNA in the CSF. After her arrival, her consciousness level worsened and she showed decerebrated posture and seizure. We immediately performed brain ventricular drainage and administered trimethoprim/sulfamethoxazole. A few days after initiation of the treatment, her mental status recovered completely without apparent sequelae. Soon after the drainage, cardiotocography showed nonreassuring fetal status, and a cesarean section was conducted immediately. A female neonate (birthweight 1246 g) was delivered with Apgar scores of 3 and 5 at 1 and 5 minutes, respectively. Zidovudine, to prevent mother‐to‐child HIV transmission, at 98 mg (2 mg/kg) was administered intravenously over 1 hour until the start of the operation and continued at 1 mg/kg/h until the end of delivery. Despite this, the neonate was proved to have HIV infection and congenital toxoplasmosis.

**Figure 2:**
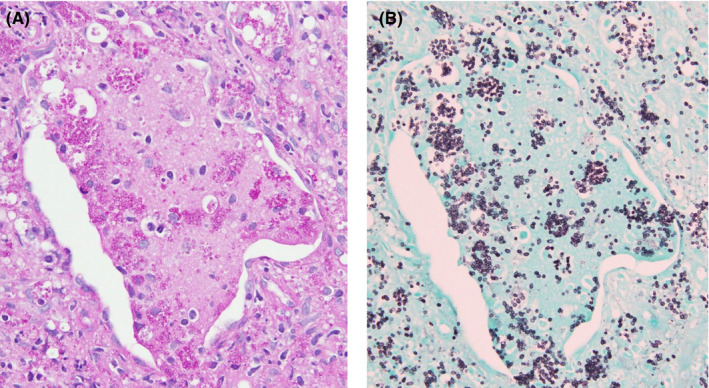
Histology transbronchial lung biopsy showing, multiple yeast colonies positive in periodic acid‐Schiff staining (A) and in Grocott staining (B). Magnification: 40×

At the same time, we intravenously administered liposomal amphotericin B (L‐AmB) at 250 mg (6 mg/kg/d) from day 1 as an empiric therapy, because cryptococcal meningitis was included in the initial differential diagnosis. The cultures of blood, urine, and CSF obtained on day 1 grew no bacteria, including acid‐fast bacilli and fungus. On day 3, we performed bronchoscopy and transbronchial lung biopsy (TBLB) in the right B4b. The histological findings of TBLB showed numerous yeast colonies positive in both periodic acid‐Schiff (Figure 2A) and Grocott staining (Figure 2B). The cultures of bronchoalveolar lavage, lung tissue, and sputum grew *Penicillium* sp. We suspected a *Penicillium* sp. infection owing to the observation of its characteristic soluble red pigment on Sabouraud dextrose agar at 25°C in the mold form and growth on the same medium at 37°C without the red pigment in its yeast form. On day 7, the sequence observed in the internal transcribed spacer (ITS) 1‐5.8S‐ITS 2 gene regions identified the pathogen to be *P marneffei*. We diagnosed the patient with pulmonary penicilliosis, who presented with a lung nodule without respiratory symptoms. On day 16, we found that *Cryptococcus neoformans* antigen was absent in both blood and CSF. Thereafter, we switched treatment from L‐AmB to oral itraconazole at 600 mg/d for three days as a loading dose, followed by 400 mg/d for pulmonary penicilliosis for 10 weeks. Chest radiography showed that the lung nodule had diminished by day 30 (Figure 1C).

On day 14, we initiated the administration of raltegravir, tenofovir, and emtricitabine, to treat HIV infection. On day 29, the CD4 T‐cell count was still low at 14/μL, despite the diminution of the HIV‐1 RNA load in the blood to 140 copies/mL. On day 46, the patient was discharged without any symptoms.

## 3 DISCUSSION

*Penicillium marneffei* infection is a common opportunistic infection in HIV‐ positive patients in South‐East Asia including Myanmar^1^, ^3^ Unfortunately, the actual epidemiological data in Myanmar are not available. Ranjana et al reported 198 HIV‐positive patients who attended hospitals between 1998 and 1999 in Manipur, India, which borders Myanmar and is ecologically and culturally similar to Myanmar. Fifty (25.3%) of those patients were recognized to be positive for *P marneffei* infection.^4^

Patients with penicilliosis present with fever, weight loss, skin lesions, lymphadenopathy, hepatomegaly, or pulmonary infiltrates.^2^ Deesomchok et al and Zhou et al reported that patients with pulmonary penicilliosis show fever (83%‐93%), cough (83%‐87%), dyspnea (75%), sputum production (40%), and hemoptysis (17%‐26%).^5^, ^6^ However, our penicilliosis case showed a lung mass but no respiratory symptoms. Reports on asymptomatic patients with penicilliosis are rare, and only two asymptomatic *P marneffei* fungemia cases have been reported.^7^, ^8^ Since asymptomatic patients may not visit a medical institution, the asymptomatic phase might be underestimated. This could be important, because the mortality of patients infected with *P marneffei* is high, unless the infection is diagnosed accurately and appropriate therapy is promptly administered. Zhou et al reported 15 penicilliosis cases, including 9 HIV‐infected patients. All 15 cases were initially misdiagnosed and correct diagnosis was made in 33 days on average. Of the 9 HIV‐infected patients, 2 died before making the correct diagnosis and 3 died during the course of therapy.^6^ Currently, many people travel worldwide; hence, there may be cases of unreported penicilliosis even in nonendemic countries.

In many cases of symptomatic pulmonary penicilliosis, successful treatment could be achieved with both AmB and itraconazole.^5^, ^9^, ^10^ In this case, we initially administered L‐AmB as an empiric therapy because cryptococcal meningitis was included in the initial diagnosis. After the nonidentification of cryptococcal antigen, we decided to continue L‐AmB treatment followed by itraconazole to treat pulmonary penicilliosis, although our patient did not have respiratory symptoms. This was because we were concerned that an untreated fungal burden of *Penicillium* could lead to immune reconstitution inflammatory syndrome (IRIS), and fungal infection could easily develop again owing to the low count of CD4 T cells. The development of IRIS has been reported to be less frequent in patients treated with L‐AmB followed by itraconazole than in those treated with itraconazole alone.^11^ In two previous reported asymptomatic *P marneffei* fungemia cases, one patient was treated with itraconazole alone and the other was not treated with any antifungal therapy but also did not develop overt penicilliosis.^7^, ^8^

In conclusion, the lack of prior documentation of an asymptomatic lung mass in penicilliosis suggests that similar cases may have been overlooked. HIV‐infected patients with abnormal findings on chest radiography, especially from South‐East Asia, *P marneffei* infection should be tested, even if patients show only a lung mass and no other respiratory symptoms.

## CONFLICT OF INTEREST

The authors have stated explicitly that there are no conflicts of interest in connection with this article.

## INFORMED CONSENT

We obtained informed consent from the patient for publication of this case report.
